# Self-Assembling Behavior of Smart Nanocomposite System: Ferroelectric Liquid Crystal Confined by Stretched Porous Polyethylene Film

**DOI:** 10.3390/nano10081498

**Published:** 2020-07-30

**Authors:** Alexej Bubnov, Alexey Bobrovsky, Ivan Rychetský, Ladislav Fekete, Věra Hamplová

**Affiliations:** 1 Institute of Physics of the Czech Academy of Sciences, Na Slovance 1999/2, 182 21 Prague, Czech Republic; rychet@fzu.cz (I.R.); fekete@fzu.cz (L.F.); hamplova@fzu.cz (V.H.); 2 Faculty of Chemistry, Moscow State University, Leninskie gory, 119992 Moscow, Russia; bbrvsky@yahoo.com

**Keywords:** nanocomposite, self-assembling behavior, nanomaterials, ferroelectric liquid crystal, smectic phase, polar order, porous polyethylene film

## Abstract

The control and prediction of soft systems exhibiting self-organization behavior can be realized by different means but still remains a highlighted task. Novel advanced nanocomposite system has been designed by filling of a stretched porous polyethylene (PE) film with pore dimensions of hundreds of nanometers by chiral ferroelectric liquid crystalline (LC) compound possessing polar self-assembling behavior. Lactic acid derivative exhibiting the paraelectric orthogonal smectic A* and the ferroelectric tilted smectic C* phases over a broad temperature range is used as a self-assembling compound. The morphology of nanocomposite film has been checked by Atomic Force Microscopy (AFM). The designed nanocomposite has been studied by polarizing optical microscopy (POM), differential scanning calorimetry (DSC), small and wide-angle X-ray scattering and broadband dielectric spectroscopy. The effect of a porous PE confinement on self-assembling, structural, and dielectric behavior of the chiral LC compound has been established and discussed. While the mesomorphic and structural properties of the nanocomposite are found not to be much influenced in comparison to that of a pure LC compound, the polar properties have been toughly suppressed by the specific confinement. Nevertheless, the electro-optic switching was clearly observed under applied electric field of low frequency (210 V, 19 Hz). The dielectric spectroscopy and X-ray results reveal that the helical structure of the ferroelectric liquid crystal inside the PE matrix is completely unwound, and the molecules are aligned along stretching direction. Obtained results demonstrate possibilities of using stretched porous polyolefins as promising matrices for the design of new nanocomposites.

## 1. Introduction

Functional composite nanomaterials are widely spread in our everyday life. During the last few years, various composite materials attract substantial attention due to their advanced properties [1,2,3,4,5,6,7,8,9] that are usually inaccessible for the single component substances. According to definition, the composite materials are those made of at least two constituent compounds with significantly different physical and/or chemical properties that, when combined, might produce a smart resulting material with advanced characteristics different from the individual components under condition that individual components remain chemically separate and distinctive within the final composite structure.

For the last few decades, self-assembling materials represent a fascinating area of dynamic research, which provides a highlighted approach for design of new structures with predefined functionality [7,10,11,12,13]. Chiral smectic liquid crystalline (LC) materials belong to one of the most exciting classes of such organic materials that are able to self-assemble in a polar layered structure of nanometer scale [11,13,14,15,16,17,18]. The self-organization structure of such compounds, and hence their favorable electro-optic, structural, and dielectric properties, can be adjusted by a proper design of molecular architecture build up from various units [15,19,20,21,22,23,24]. Chiral liquid crystals derived from the lactic acid are intensively investigated due to definite advantages [19,25,26] with respect to other types of chiral molecular structures: chemical stability - no aging, reasonably low price, and a comprehensive variety of conventional and frustrated nematic and smectic phases stable in a broad temperature range. LC compounds with chiral part based on lactate group are actively used as: (i) chiral dopants while designing binary [27,28,29] and multicomponent functional mixtures [30,31]; (ii) reactive mesogens for macromolecular compounds used as side-chains for polymers [32,33,34] and elastomers [35,36]; (iii) functional dopants for organic photovoltaic cells [37,38,39] and matrices [40,41,42] for design of nanocomposite systems; and (iv) source of chirality for photosensitive low molar mass [21,43,44,45] and macromolecular [20,34,46,47,48] materials.

One of the main problems related to applicability of chiral LC compounds exhibiting the synclinic and anticlinic phases, which is not fully solved until now, is the mechanical control and stability; the stability to mechanical shock can be taken as a specific example. This stability requirement affects a lot the feasibility of practical commercial applications based on polar LC compounds [11,15]. It is unfavorable and one of the main reasons why, about two decades ago, the commercial utilization of the ferroelectric LC displays was failed and the nematic/cholesteric materials almost fully gain the market. Mechanical stabilization of the self-organizing systems and structures by various polymeric treatment is under continuous and intense investigations [49,50,51]. Polymer dispersed liquid crystals are composite compounds formed by micron-sized droplets of liquid crystal embedded in a solid polymer matrix [52,53,54,55,56] attracted great attention of researchers due to their applicability as light shutters, switchable windows, displays, and other electro-optical devices.

In general, while designing new self-assembling structures, porous materials can be used as a smart media which allows to stabilize mechanically the desired properties [57,58,59,60,61,62]. Specifically, porous macromolecular materials have attracted durable attention of the scientific community [58,63,64,65,66] due to their functionality and possibility of mechanical control. Nevertheless, this is quite challenging, for a general task to predict and keep under control [67,68,69] the desired properties of the self-assembling materials. Several attempts for investigation of stretched porous polyethylene (PE) filled with LC compounds have been already done [56,70,71,72,73]. It has been shown that porous stretched PE-based nanocomposites filled with photosensitive LC compounds can be used as light-sensitive actuators [74,75], advanced optical and opto-fluidic devices [70,76], nanofiltration membranes [65], and technologically attractive material for random lasing [77]. One of the most important features of the porous stretched PE is the capability to align embedded LC molecules along the stretching direction [55,73]. This makes porous polyolefin matrices very promising for the design of novel functional nanocomposite materials.

However, still a lot of open questions remains regarding the basic interaction of porous media (used as a polymeric flexible matrix) and LC molecules, specifically while dealing with those organized in a polar smectic structure. Electro-optical behavior of nanocomposite system made of porous stretched PE film filled by multicomponent ferroelectric LC mixture has been studied [73] and ferroelectric switching of such composites was demonstrated for the first time. In order to effectively tune such a system and to reach the desired properties, it is necessary to gain deeper knowledge on interactions between the porous media and self-organized polar matter [73].

The main objective of the work is to establish the mesomorphic, dielectric, and structural behavior of a novel PE-ferroelectric liquid crystalline (FLC) nanocomposite system build-up of the porous stretched PE matrix filled by the ferroelectric liquid crystalline (FLC) compound and to contribute to better understanding of interactions between the stretched porous PE and FLC compound taking place in such a system.

## 2. Materials and Methods

The nanocomposite samples of stretched porous PE film filled by the lactic acid derivative forming the ferroelectric smectic phase over a reasonably broad temperature region was investigated. The details on used methods, original compounds, resulted nanocomposite material, and their basic characteristics are presented in this section.

### 2.1. Microporous Stretched Polyethylene Film

**Figure 1:**
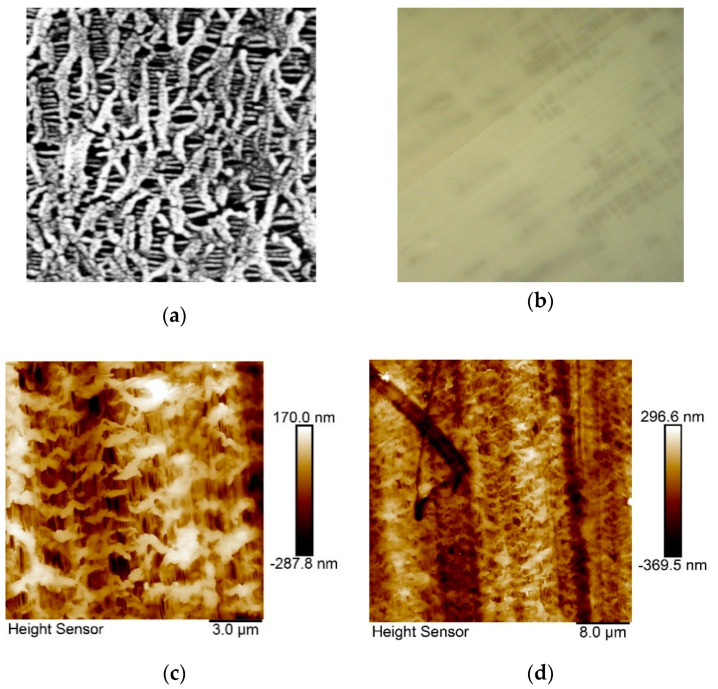
Topographical images of porous polyethylene (PE) film obtained at room temperature: (**a**) by the Scanning Electron Microscopy (SEM) (width of the photo is ~10 µm); (**b**) by the polarizing optical microscopy (POM) under crossed polarizers (width of the photo is ~250 µm) and by Atomic Force Microscopy (AFM) (**c**),(**d**) at difference places of the sample under resolution, as indicated. The stretching direction for: (**a**) is horizontal; (**c**,**d**) vertical.

The microporous stretched polyethylene (PE) film was obtained from commercially available low-density PE (M*_w_* = 1.7 × 10^5^, M*_w_*/M*_n_* = 5–6, T_m_ = 132 °C) according to specific procedure as described [56,57]. Porous PE films preparation consists of several steps: extrusion of the PE melt through a flat-slit die followed by annealing at isometric conditions, uniaxial stretching at room temperature (stage of pores formation), and thermal fixation. The main controllable parameters predetermining the PE porosity and sizes of the pores are the spinneret drawing ratio during extrusion (60 in our case), annealing temperature (130 °C), and degree of the uniaxial stretching (250%). Further details of PE films processing during pores formation is described in detail in Ref. [56,57]. During extrusion and stretching, the porous structure (with the pore width of 50–600 nm and pore length of 1000–2000 nm) was formed. The thickness of the PE film was 17.0 ± 0.3 µm, and pores occupy approximately 40–50% of volume. Microphotographs of porous PE film obtained by the Scanning Electron Microscopy (SEM) and by Polarizing Optical Microscopy (POM) under cross polarizers are presented on Figure 1a,b, respectively, and the topographical Atomic Force Microscopy (AFM) images at two different places of the used sample, with resolution as indicated, are presented on Figure 1c,d. The porous structure of the stretched PE film is clearly confirmed by both the SEM and AFM techniques.

#### Ferroelectric Liquid Crystal

**Figure 2:**
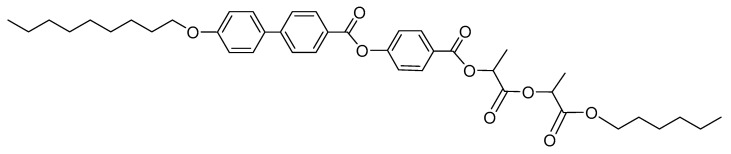
Chemical structure of ferroelectric smectic compound, namely (4-(((1-((1-(hexyloxy)-1-oxopropan-2-yl)oxy)-1-oxopropan-2-yl)oxy)carbonyl)phenyl 4′-(nonyloxy)-[1,1′-biphenyl]-4-carboxylate), used for the resulting polyethylene (PE)-ferroelectric liquid crystalline (FLC) nanocomposite.

Lactic acid derivative, namely 4-(((1-((1-(hexyloxy)-1-oxopropan-2-yl)oxy)-1-oxopropan-2-yl)oxy)carbonyl)phenyl 4′-(nonyloxy)-[1,1′-biphenyl]-4-carboxylate, possessing specific mesomorphic properties, was been selected for this study. The synthetic route and mesomorphic properties of the FLC compound (denoted as ZLL 9/6 [78]) used for design of PE-FLC nanocomposite film were described recently [78,79]. The general chemical formula of the FLC compound is presented on Figure 2. Sequence of mesophases was determined by the POM observations and was checked by DSC. On cooling from the isotropic phase (Iso), this compound possesses (at 113 °C) the paraelectric orthogonal smectic A* (SmA*) phase (down to 94 °C), followed by a broad ferroelectric tilted smectic C* (SmC*) phase (down to 42 °C), which was found partially monotropic as the melting point is detected at 79 °C. A narrow (several degrees broad) and fully monotropic (i.e., the mesophase was detected on cooling run only) tilted hexatic (Hex*) phase was detected before the crystallization (Cr) onset.

### 2.2. Experimental Methods

The nanocomposite based on porous PE film filled with FLC compound was prepared according to procedure described below. Porous stretched PE film (supplied by professor G.K. Elyashevitch) was heated up and filled by the FLC compound in the isotropic phase (at 115 °C) by means of capillary action; liquid crystal was sucked by the porous film by means of capillary action, and the pores were filled completely; afterwards, the filled nanocomposite film was cooled down to room temperature. The excess of the FLC compound was cleaned up by a solvent (acetone) from the surfaces, and the glasses with transparent Indium Tin Oxide (ITO) conductive layers were attached to the film; then, they were stacked together by a special glue based on phenol formaldehyde resin. In order to perform the morphology study, Atomic Force Microscopy (AFM) measurements have been executed using a Bruker Dimension Icon microscope (Bruker, Santa Barbara, CA, USA), working for material-air surface in the PeakForce mode at the room temperature. Cantilevers with a low spring constant, *k* = 0.4 N m^−1^ were used, with the resonant frequency in a range of 70–80 kHz. AFM images of resolution 512 × 512 points were obtained; the images were taken in several different spots of the sample and areas with several different size and resolution; the AFM measurements were done at room temperature.

The measurement cells for mesomorphic, electro-optic, and dielectric studies of pure FLC compound were prepared by a commonly used method. The FLC compound was filled by capillary action into a homemade glass cell possessing on the inner side the ITO transparent electrodes and polyimide layers unidirectionally rubbed, which ensured planar (bookshelf) geometry. The sample thickness was defined by 17 µm thick mylar sheets.

The mesomorphic properties of pure FLC compound and resulting nanocomposite films, namely the sequence of phases and phase transition temperatures, were determined on cooling in polarizing optical microscope by observation of the characteristic textures and their changes. A LINKAM LTS E350 heating/cooling stage with TMS 93 temperature programmer was used for temperature control, which enabled temperature stabilization within ± 0.1 K. Phase transition temperatures and melting points (m.p.) were determined by the differential scanning calorimetry (DSC-Perkin-Elmer DSC8000) on samples of 8–10 mg, hermetically sealed in aluminium pans, on cooling/heating runs (5 K min^−1^ rate) in a nitrogen atmosphere. The temperature was calibrated on extrapolated onsets of melting points of water, indium, and zinc.

Values of spontaneous polarization, *P_s_*, were determined from the switching current profile detected by oscilloscope Tektronix DPO 4034 (Tektronix, Beaverton, OR, USA). Electric field of triangular modulation at frequency of 19 Hz was used.

The frequency dispersion of complex permittivity (ε* = ε′ − iε″) was measured on cooling using a Schlumberger 1260 impedance analyzer in the frequency range of 1 Hz–1 MHz, keeping the temperature stable (within ± 0.1 K) during the frequency sweep. Dielectric measurements at fixed frequency have been done under d.c. bias voltage of 0–20 V. Cells of 17 µm thick were used for the broad-band dielectric spectroscopy.

The small angle X-ray scattering (SAXS) was performed with Ni-filtered CuK_α_ radiation (wavelength λ = 1.5418 Å) on non-aligned samples (filled into Mark capillary tubes of 0.7 mm diameter) using a Kratky compact camera (A. Paar) equipped with a temperature controller and a one-dimensional electronic detector (M. Braun); temperature was controlled within 0.1 K. The smectic layer spacing, d, was determined using Bragg’s law *nλ* = 2*d*sinθ, where *n* is a positive integer and d is calculated from the position of the small angle (θ = 0.2^o^–4.5^o^) diffraction peaks. The wide-angle X-ray scattering (WAXS) was performed with a Bruker AXS NanoSTAR device (wavelength λ = 1.5418 Å) equipped with a temperature control unit, magnetic field for sample alignment, and two-dimensional detector.

## 3. Results

This section contains the experimental results obtained on PE-FLC nanocomposite obtained by POM, DSC, electro-optics, SAXS, WAXS, and broadband dielectric spectroscopy. The discussion on significant permittivity decrease obtained for PE-FLC nanocomposite and partial suppression of the polar smectic order are presented.

### 3.1. Mesomorphic Properties and Switching Behavior

The mesomorphic behavior of pure FLC compounds and resulting PE-FLC nanocomposite was studied by the texture observations in POM; the phase transition temperatures were precisely verified by the DSC. Table 1 summarizes the obtained data on the mesomorphic behavior. The sequence of mesophases for both the pure FLC and PE-FLC nanocomposite remains unchanged; on cooling from the isotropic (Iso) phase, the paraelectric orthogonal SmA* and the ferroelectric tilted SmC* phases were clearly detected; the Iso-SmA* phase transition is of the first order and the SmA*-SmC* phase transition is of the second order. In addition, there is a very narrow hexagonal phase before the onset of the crystallization, i.e., the crystal phase (Cr). However, there is a considerable difference in the Iso-SmA* and SmA*-SmC* phase transition temperatures. In case of the PE-FLC nanocomposite, the above-mentioned phase transition temperatures were found to be shifted down on both the cooling and heating runs; this is quite common mesomorphic behavior when studying pure LC compounds, as well as LC nanocomposites [42,73]. The difference in the phase transition temperatures observed for pure FLC compound and PE-FLC composite was also confirmed by dielectric spectroscopy and SAXS measurements.

**Figure 3:**
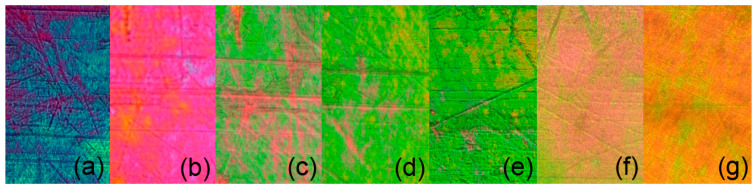
Optical microphotographs of PE-FLC nanocomposite obtained by POM on cooling, indicating strong change of birefringence: (**a**) the isotropic phase (Iso)-smectic A* (SmA*) phase transition at 105 °C, (**b**) the SmA* phase at 100 °C, (**c**) the SmA*-smectic C* (SmC*) phase transition at 88 °C, (**d**–**f**) the SmC* phase (at 86 °C, 60 °C, and 45 °C, respectively), (**g**) the Hex phase (at 40 °C). Width of each sub-microphotograph is ~300 µm.

Microphotographs of PE-FLC nanocomposite film obtained by POM (17 µm thick film) on cooling from the isotropic down to low temperatures are presented in Figure 3a–g. Strong change of the birefringence with temperature was clearly observed.

**Figure 4:**
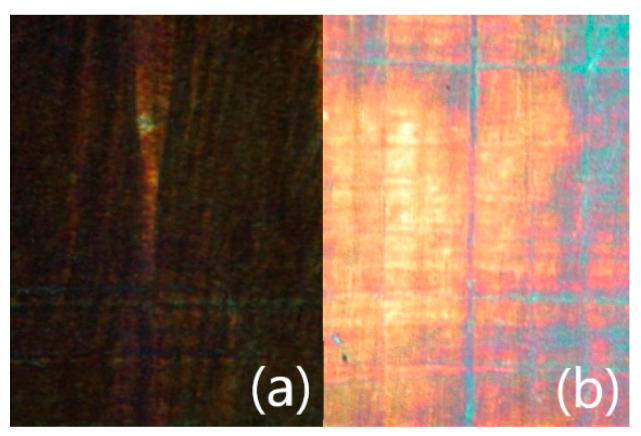
Microphotographs of textures obtained by POM in the SmC* phase (at 75.0 °C) of the PE-FLC nanocomposite film (17 µm thick) after alignment by the a.c. electric field of low frequency (10–20 Hz, 40 kV cm^−1^ applied for 5–30 min.): (**a**) the angle between the polarizer plane and main direction of porous long axes is 45° (dark state) and (**b**) the angle between the polarizer plane and main direction of porous long axes is 0° (bright state). Width of each sub-microphotograph is ~250 µm.

For the pure FLC compound, values of spontaneous polarization (P_s_) reach ~100 nC cm^−2^ at saturation, which is quite typical values for the chiral LC homologues belonging to the ZLL-series [19,78]. The electro-optic response of PE-FLC nanocomposite has been detected at very high voltages of *V*_pp_ = 210 V (19 Hz). The electro-optic switching was clearly observed under applied electric field of low frequency. The “bright” and “dark” states are shown on Figure 4. However, while trying to estimate the spontaneous polarization values, no current bumps that could be associated with spontaneous polarization were detected under this field. This effect can be explained by the suppressing of the polar order of the ferroelectric SmC* phase confined by the inner boundaries of the stretched porous PE film.

### 3.2. Structural Properties

**Figure 5:**
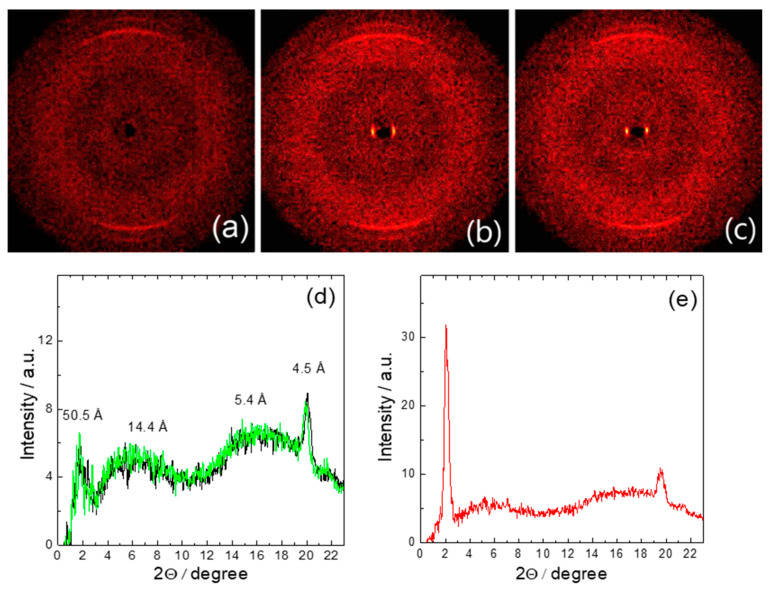
Two-dimensional-X-ray patterns for: (**a**) pure stretched PE film (30 °C); (**b**) stretched PE-FLC nanocomposite in the SmC* phase (~70 °C); (**c**) stretched PE-FLC nanocomposite in the SmA* phase (~106 °C). For (**a**–**c**), stretching direction is horizontal. The wide-angle X-ray scattering (WAXS) intensity profile measured for (**d**) the pure stretched PE film (at 30 °C and 90 °C, black and green curves, respectively) and (**e**) for the PE-FLC nanocomposite (at 70 °C).

Figure 5 shows the WAXS results, specifically the 2D-X-ray patterns obtained for stretched porous PE film (Figure 5a), for PE-FLC nanocomposite in the ferroelectric SmC* (Figure 5b) and in the paraelectric SmA* phases (Figure 5c) at indicated temperatures. The corresponding WAXS intensity profiles are shown on Figure 5d for pure stretched PE film (at 30 °C and 90 °C) and on Figure 5e for stretched PE-FLC nanocomposite (at 70 °C). WAXS results clearly indicate the alignment of the long molecular axis of the FLC material along the stretching direction of the studied porous PE film.

**Figure 6:**
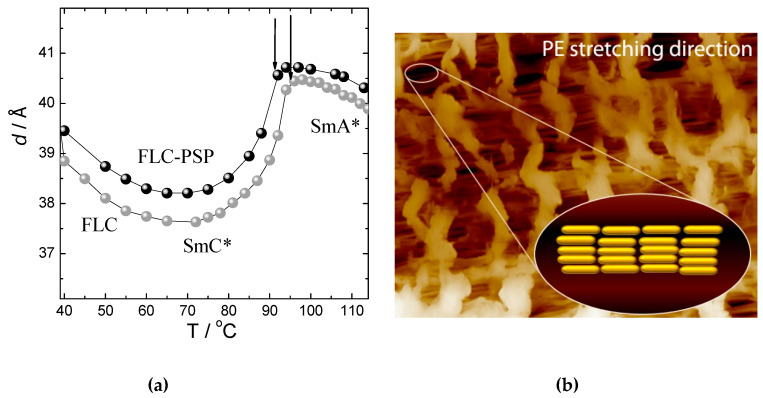
Temperature dependence (**a**) of the smectic layer spacing, *d*, for pure FLC compound (grey circles) and for the PE-FLC nanocomposite (black circles) measured on cooling. Vertical arrows indicate the SmA*-SmC* phase transition. The measurement error approximately corresponds to the size of symbols. (**b**) Schematic cartoon of the molecular orientation in SmA* phase inside the pores of the stretched PE film; stretching direction is horizontal; rods stacked in layers represent the FLC molecules oriented along the stretching direction of the PE film.

Temperature dependence of the smectic layer spacing, d, for pure FLC compound and for PE-FLC nanocomposite measured on cooling in the temperature range of the SmA* and the SmC* phases is shown on Figure 6a. The cartoon showing that the long molecular axis is oriented along the stretching direction of the porous PE film is presented in Figure 6b. The hexatic Hex* phase (might be either SmI* or SmF*) was not characterized due to a very narrow temperature range and its fully monotropic character (i.e., this is a fully overcooled phase). While approaching the SmA*-SmC* phase transition on cooling, pure FLC material exhibited a slight increase of the smectic layer spacing values (see Figure 6a), which is obviously due to stretching of aliphatic molecular chains with decrease of temperature. The same explanation is valid also for a slight increase of d values at lower temperatures in the SmC* phase. Nevertheless, a strong decrease of the smectic layer spacing values close below the SmA*-SmC* phase transition clearly related to the increase of the tilt angle of molecules with respect to the smectic layer normal. Overall, qualitatively similar behavior of the smectic layer spacing was observed for the PE-FLC nanocomposite; a slight shift-up of the respective curve can be explained by a difference in the SAXS setup calibration. The value of the tilt angle determined from the SAXS data [79] for the pure FLC compound is 22.2 degrees at 20 °C below the SmA*-SmC* phase transition temperature. Nevertheless, the results of X-ray studies show that the layered structure of the smectic phases for the PE-FLC nanocomposite is fully preserved as it was determined for the pure FLC compound. However, there is a slight decrease in the SmA*-SmC* phase transition temperature for the PE-FLC nanocomposite with respect to that of the pure FLC compound.

**Figure 7:**
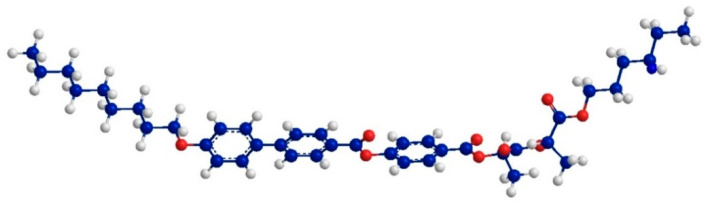
Conformation of the FLC molecule used for the PE-FLC nanocomposite after energy minimization using MOPAC/AM1 method. The length of the most extended conformer is 38.5 Å.

The MOPAC/AM1 model was used to calculate the length of FLC molecules in the energy optimized conformation. The molecular structure with the principal axis of minimum moment of inertia (“long molecular axis”) is presented in Figure 7. Taking into account the most extended conformer, the length of molecule, L, is 38.5 Å. This value appropriately correlates with the layer spacing values obtained within the orthogonal SmA* phase (see Figure 6a); the difference can be related to the non-perfect orientational order [79].

**Figure 8:**
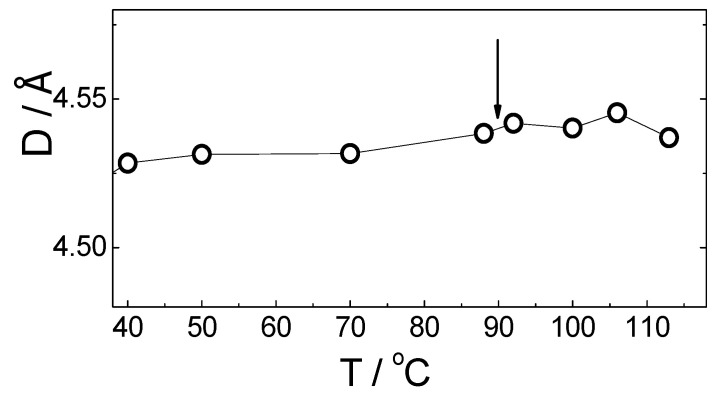
Temperature dependence of the average distance, D, between the long axis of the FLC molecules measured by WAXS on cooling for the PE-FLC nanocomposite. Vertical arrow indicates the SmA*-SmC* phase transition temperature.

Temperature dependence of the average distance, D, between the long axis of FLC molecules (i.e., intermolecular distance) measured by WAXS on cooling for the PE-FLC nanocomposite is presented on Figure 8. There is a continuous decrease of the intermolecular distance of the long molecular axis on cooling, indicating the increase of the positional order; no anomalies in intermolecular distance values are observed at the SmA*-SmC* phase transition (see Figure 8).

### 3.3. Dielectric Properties

**Figure 9:**
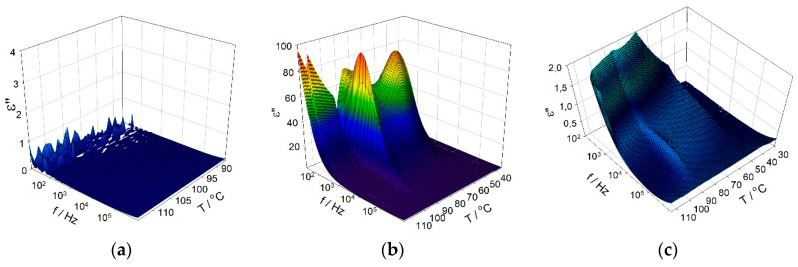
Three-dimensional-plots of the imaginary part of complex permittivity versus frequency and temperature measured on cooling for: the pure PE film (**a**), pure FLC compound (**b**), and stretched porous PE-FLC nanocomposite (**c**).

Broadband dielectric spectroscopy was done on empty stretched porous PE film (Figure 9a), pure FLC compound (Figure 9b), and on the resulting PE-FLC nanocomposite (Figure 9c). The imaginary parts, ε″, of complex permittivity for all three above-mentioned systems versus temperature and versus frequency are presented in Figure 9a–c as an illustrative result to compare the dielectric response and to check the ferroelectric character of the polar smectic phase. For the pure PE film, no response resulting from the collective behavior was detected (see Figure 9a). For pure FLC compound (see Figure 9b), the dielectric spectra obtained within the whole temperature range of the paraelectric SmA* and the ferroelectric SmC* phases at zero bias electric field reveal a strong contribution of the Goldstone mode (the relaxation mode related to azimuthal fluctuations of the molecules in the smectic layer), especially in the SmC* phase. In the vicinity of the SmA*-SmC* phase transition, a collective mode related to molecular fluctuations in the tilt magnitude, so-called soft mode, was observed. This behavior fully confirms the paraelectric character of the orthogonal SmA* phase and the ferroelectric character of the tilted SmC* phase detected for pure FLC compound. This is quite typical dielectric behavior of the soft and Goldstone modes as it was shown by various authors before [19,30,31,45]. The dielectric behavior of the PE-FLC nanocomposite is presented on Figure 9c. The detected collective relaxation processes look completely different if compared to that of pure FLC compound (see Figure 9c). In the ferroelectric phase, the value of dielectric constant for the PE-FLC nanocomposite is approximately 10^2^ times lower than that for pure FLC compound. This indicates that the structure of the FLC confined inside the PE matrix differs from the bulk FLC. Recently, it has been shown [80,81] that, in the confined FLC, there are two important but conflicting influences. The boundary conditions due to the anchoring of polar molecules at the surfaces that prefer the twisted structure, while the interactions between molecules favor the helical structure [80,81]. Both effects can also coexist, but only with the help of the dechiralization defects. When the size of the confinement is comparable with the helix periodicity, the helical structure starts to be completely unwound and the pure twisted structure occurs. The boundary conditions also bring about the internal depolarizing fields, which can be responsible for drop down of the dielectric constant [80,81].

**Figure 10:**
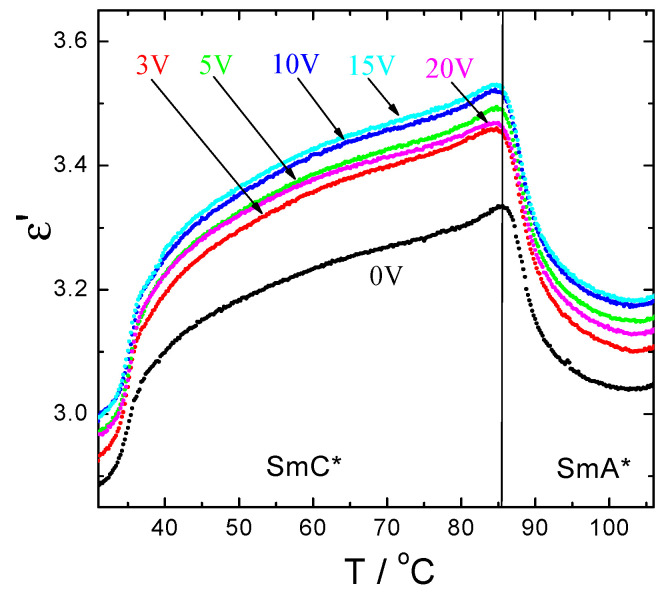
Temperature dependence of real part of complex permittivity measured (at frequency of 2 kHz) on cooling for PE-FLC nanocomposite under d.c. bias field as indicated.

Temperature dependence of real part of complex permittivity measured at 2 kHz on cooling for PE-FLC nanocomposite under d.c. bias field is presented in Figure 10. The applied d.c. bias electric field (as indicated) does not influence much the value of dielectric constant in the ferroelectric SmC* phase, which also confirms the absence of the helical structure for the PE-FLC nanocomposite.

### 3.4. Discussion on Significant Permittivity Reduction of PE-FLC Nanocomposite

As it has been shown in the previous subsection, the dielectric properties of the ferroelectric LC compound confined in the stretched porous PE film are strongly affected by the boundaries [80,81], i.e., by the inner surface of PE film pores. The decrease of permittivity values, while comparing the bulk FLC sample and stretched porous PE film filled with FLC, is quite huge and can be expressed as their ratio:

(1) $$\frac{\varepsilon }{\varepsilon_{FLC}}=\frac{1+\chi }{1+\chi_{FLC}}\approx 10^{-2}.$$

Assuming that the typical size of PE matrix pores, *d_p_*, is of order micrometers $d_{p}\approx 1\mu m$ and the FLC compound is confined inside pores, the helical structure of the chiral tilted SmC* phase is expected to be almost completely unwound. However, due to the strong anchoring of the FLC molecules at pore boundaries, the FLC compound is expected to exhibit a twisted structure while placed inside the pores [80,81]. In such case, in contrast with the ideal helical FLC structure [15], the bound electrical charges are induced, resulting in quite strong internal (depolarising) fields. These depolarizing fields are not fully compensated since oscillating external electric field is applied.

For qualitative estimation let us consider a twisted structure in the layer of the thickness, *d_p_*. Then, the susceptibility of the system can be expressed (in SI units) as in Reference [81]:

(2) $$\chi =\frac{\chi_{FLC}}{{\left(\frac{p}{2d_{p}}\right)}^{2}+\pi L\chi_{FLC}},$$

where $\chi_{FLC}$ is the susceptibility of an ideal helical ferroelectric liquid crystal structure, *p* is its helical pitch length, and 0≤L≤1 is a depolarizing factor. A zero value of the depolarizing factor, *L* = 0, corresponds to the full charge compensation.

When the ratio *p*/*d_p_* ~ 1, which is exactly our case, and using the susceptibility decrease described by relation (1) with the relation (2), the depolarizing factor, *L*, can be estimated as: L ≈ 0.1–0.2 (it has been assumed that ε≈3 and $\varepsilon_{FLC}\approx 250$). According to the explanation shown above, it can be summarized that the dielectric response of the PE-FLC nanocomposite is highly sensitive to uncompensated electrical charges and even their small amount might explain a significant reduction of permittivity values while comparing the pure FLC sample and the stretched porous PE film filled with the FLC material.

## 4. Conclusions

Properties of the nanocomposite thin film composed of porous stretched PE film filled with the lactic acid derivative possessing the ferroelectric SmC* phase (PE-FLC) were studied by several experimental techniques. For the PE-FLC nanocomposite, there was a slight decrease of the Iso-SmA* and SmA*-SmC* phase transition temperatures with respect to those of pure FLC compound; this effect wasclearly observed on both the cooling and heating runs and was not related to the temperature hysteresis peculiar to LCs, i.e., the difference of the phase transition temperatures obtained on heating and cooling runs due to non-zero heating cooling rates. In the ferroelectric phase, the electro-optical response on applied electric field was clearly observed. However, ferroelectric switching was not detected up to 10 kV/cm electric field. According to SAXS results, the layered structure of the paraelectric and ferroelectric smectic phases of the PE-FLC nanocomposite fully corresponds to that of the pure FLC compound. The capability of the porous stretched PE to align embedded FLC molecules along stretching direction was confirmed. This makes porous polyolefin matrices very promising for design of novel functional nanocomposite materials. Quite high permittivity values obtained in the ferroelectric phase of pure FLC, which is related to the contribution of the Goldstone mode, was strongly suppressed in the case of PE-FLC nanocomposite film. This clearly indicates the suppressing of the molecular reorientation for this specific self-organized nanocomposite system. This effect can be attributed to the size-effect observed in the dielectric response of FLC compound confined inside micrometric regions and to the internal electric fields always existing at the micro-region boundaries.

The PE-FLC composite materials can be potentially used for various advanced applications, taking into account definite advantages of porous stretched PE films, namely a simple manufacturing, high chemical and mechanical stability, low production costs, availability of raw material, and unique properties of low molar mass chiral liquid crystals with polar self-assembling behavior. For example, addition of photosensitive dyes to FLC substance enables us to achieve dual electro- and photoinduced control of the optical properties of the composites that can be promising for application in photonics and optoelectronics. Further studies of such PE-FLC nanocomposite systems are in progress now and will be presented elsewhere.

## Figures and Tables

**Table 1:** Sequence of phases determined by POM and phase transition temperatures (°C) measured by differential scanning calorimetry (DSC) on cooling (5 K min^−1^) for the pure FLC compound and resulting PE-FLC nanocomposite. Symbol <●> stands for the presence of the mesophase.

Material	Cr	°C	Hex*	°C	SmC*	°C	SmA*	°C	Iso
Pure FLC	●	39	●	42	●	94	●	113	●
PE-FLC composite	●	39	●	42	●	88	●	105	●
